# Evaluation of Anterior Cervical Reconstruction with Titanium Mesh Cages versus Nano-Hydroxyapatite/Polyamide66 Cages after 1- or 2-Level Corpectomy for Multilevel Cervical Spondylotic Myelopathy: A Retrospective Study of 117 Patients

**DOI:** 10.1371/journal.pone.0096265

**Published:** 2014-05-02

**Authors:** Yuan Zhang, Zhengxue Quan, Zenghui Zhao, Xiaoji Luo, Ke Tang, Jie Li, Xu Zhou, Dianming Jiang

**Affiliations:** Department of Orthopedic Surgery, The First Affiliated Hospital of Chongqing Medical University, Chongqing, China; Toronto Western Hospital, Canada

## Abstract

**Objective:**

To retrospectively compare the efficacy of the titanium mesh cage (TMC) and the nano-hydroxyapatite/polyamide66 cage (n-HA/PA66 cage) for 1- or 2-level anterior cervical corpectomy and fusion (ACCF) to treat multilevel cervical spondylotic myelopathy (MCSM).

**Methods:**

A total of 117 consecutive patients with MCSM who underwent 1- or 2-level ACCF using a TMC or an n-HA/PA66 cage were studied retrospectively at a mean follow-up of 45.28±12.83 months. The patients were divided into four groups according to the level of corpectomy (1- or 2-level corpectomy) and cage type used (TMC or n-HA/PA66 cage). Clinical and radiological parameters were used to evaluate outcomes.

**Results:**

At the one-year follow-up, the fusion rate in the n-HA/PA66 group was higher, albeit non-significantly, than that in the TMC group for both 1- and 2-level ACCF, but the fusion rates of the procedures were almost equal at the final follow-up. The incidence of cage subsidence at the final follow-up was significantly higher in the TMC group than in the n-HA/PA66 group for the 1-level ACCF (24% vs. 4%, p = 0.01), and the difference was greater for the 2-level ACCF between the TMC group and the n-HA/PA66 group (38% vs. 5%, p = 0.01). Meanwhile, a much greater loss of fused height was observed in the TMC group compared with the n-HA/PA66 group for both the 1- and 2-level ACCF. All four groups demonstrated increases in C2-C7 Cobb angle and JOA scores and decreases in VAS at the final follow-up compared with preoperative values.

**Conclusion:**

The lower incidence of cage subsidence, better maintenance of the height of the fused segment and similar excellent bony fusion indicate that the n-HA/PA66 cage may be a superior alternative to the TMC for cervical reconstruction after cervical corpectomy, in particular for 2-level ACCF.

## Introduction

Several surgical techniques have been suggested for the treatment of multilevel cervical spondylotic myelopathy (MCSM). However, the optimal surgical procedure remains controversial [1], [2], [3], [4]. Both anterior and posterior approaches have been reported with satisfactory clinical outcomes [5], [6], [7]. Full decompression of the spinal cord and stable reconstruction of cervical alignment are the two critical aims of this surgery [5]. Based on increased etiological data, recent reports have proposed that the compression of the spinal cord in MCSM most likely originates from anterior regions, such as cervical discs and osteophytes [8], [9], suggesting that anterior procedures may be more reasonable for MCSM. Without the limitation of the disc levels, anterior cervical corpectomy and fusion (ACCF) is considered a favorable option compared with anterior cervical discectomy and fusion (ACDF) among the anterior techniques [10]. More importantly, ACCF can remove almost all pathologies that cause spinal cord compression, such as prolapsed discs, osteophytes and ossified posterior longitudinal ligament (OPLL) [11].

**Figure 1:**
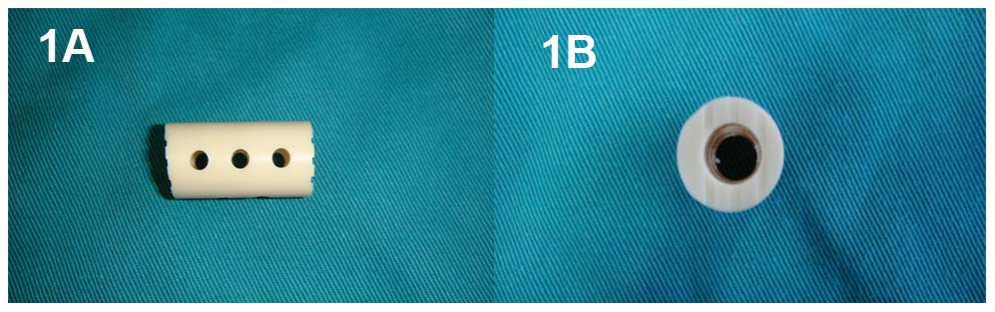
Photographs of lateral (1A) and superior (1B) views of the nano-hydroxyapatite/polyamide66 cage.

The reconstruction of the cervical spine is relatively important following corpectomy-mediated decompression. Using auto-grafts harvested from the iliac crest or fibula for fusion has been considered the “gold standard” for anterior cervical column reconstruction. Unfortunately, donor-site complications remain [12]. Allografts can be used to avoid the morbidity associated with autograft harvest, but this technique decreases the rate of arthrodesis and increases the rate of graft collapse [13]. Titanium mesh cages (TMCs) filled with local cancellous bone autografts have been used for decades for cervical reconstruction after corpectomy [9], [14], with advantages including few donor-site complications, early biomechanical stabilization, a short operation duration and high fusion rates (range: 97%–100%); however, the inevitable complication of subsidence and other disadvantages, including stress shielding and radiopacity also occur [15], [16], [17], [18], [19]. The nano-hydroxyapatite/polyamide66 cage (nano-HA/PA66 cage) is a hollow cylinder manufactured of the n-HA/PA66 composite (Figure 1). The use of this cage filled with autograft has been reported for anterior cervical reconstruction in recent years, with satisfactory clinical outcomes [20], [21]. Few studies have compared the outcomes of TMCs and n-HA/PA66 cages. The aim of the present study was to comparatively assess the clinical outcomes of TMCs vs. n-HA/PA66 cages for MCSM after 1- or 2-level corpectomy to provide a basis for selecting the appropriate method for reconstructing the cervical spine.

## Materials and Methods

This study was approved by the Institutional Review Board of the First Affiliated Hospital of Chongqing Medical University, and all aspects of the study comply with the Declaration of Helsinki. The Institutional Review Board of the First Affiliated Hospital of Chongqing Medical University also waived the requirement for patient consent because this study was retrospective, the data were analyzed anonymously and patient care was not affected by the study. Between June 2006 and December 2010, a total of 117 consecutive patients (65 males and 52 females) who underwent ACCF for MCSM by a senior surgeon (QUAN) were evaluated retrospectively. All patients presented with myelopathy prior to the operation, and magnetic resonance images confirmed MCSM diagnoses. Patients with cervical trauma, infections, tumors, rheumatoid arthritis, congenital deformities, severe osteoporosis or previous cervical spine surgery were excluded from our study.

All patients underwent a 1- or 2-level corpectomy, based on the level of the lesion, followed by cervical reconstruction with a TMC (Medtronic Sofamor Danek, Memphis, TN, USA) or an n-HA/PA66 cage (Sichuan National Nanotechnology Co., Ltd., Chengdu, China). The n-HA/PA66 cages were designed and fabricated by the Institute of Materials Science and Technology, Sichuan University, and our department, and they have been approved for clinical use since 2005 by the State Drug and Food Administration of China. The n-HA/PA66 cages have an 8- to 14-mm outer diameter and a 3- to 8-mm inner diameter and are of appropriate length for clinical utilization; each cage has grooves at each end to increase the friction between the cage and vertebral endplates and several 2-mm holes in the wall of the cage [20], [22]. We divided the patients into four groups based on the number of levels fused (1- or 2-level ACCF) and cage selection (TMC or n-HA/PA66 cage).

All surgeries were performed using a right-sided anterior cervical approach. After accurate exposure of the surgical region, a Caspar screw distraction was used for adequate distraction. Following the discectomy at the cephalic and caudal level of the lesion segment, 1- or 2-level corpectomies were performed using a Kerrison rongeur. Hypertrophic osteophytes and the posterior longitudinal ligament were removed in every case to ensure that the dura mater was widely exposed. The adjacent cartilage endplates were removed as fully as possible, and the bony endplates were preserved. An appropriately sized TMC or n-HA/PA66 cage was then prepared, filled with autogenous cancellous bone from the resected vertebra and then implanted into the intervertebral space after corpectomy using a titanium anterior cervical plate for internal fixation. All patients were instructed to wear a cervical collar for six weeks postoperatively.

The surgery time, blood loss and hospital stay were recorded. Clinical and radiological follow-ups were conducted immediately and at three months, six months and one year after surgery and then annually thereafter. The Japanese Orthopedic Association (JOA) score was used to assess neurologic status, and a 10-point visual analogue scale (VAS) was used to grade neck pain. The anteroposterior, neutral lateral and flexion/extension lateral cervical plain radiographs at preoperative, immediate postoperative, 1-year follow-up and the final follow-up were examined to assess radiologic parameters, including the height of the fused segment, the loss of height of the fused segment, cervical sagittal alignment and fusion status. The distance between the midpoint of the cephalic endplate and the caudal endplate of the fused segment was measured in millimeter (mm) and used as the height of the fused segment using Carestream software (Carestream Health, Inc. Toronto, Canada). Loss of height of the fused segment was defined as a reduction in height of the fused segment from the immediate postoperative period to follow-ups, and subsidence was defined as a loss of height of greater than 3 mm [23]. Cervical sagittal alignment was defined as the Cobb angle formed between the lower endplates of C2 and C7 on neutral lateral cervical plain film [24]. Bony fusion was defined as the trabeculation and bridging between the cage and adjacent endplates and the absence of motion between spinous processes upon flexion/extension in the fused segments. Three-dimensional computed tomography (3D-CT) scans were taken to further confirm the fusion status by observing the trabeculation between the autogenous cancellous graft and adjacent endplates [25].

Statistical analyses were performed using SPSS (version 16.0, SPSS Inc., Chicago, IL). Quantitative data are presented as the mean ± standard deviation. Repeated measures ANOVA was used for statistical analyses of differences in mean values, and the Chi-squared test was used for categorical data. The threshold for statistical significance was set to p<0.05.

## Results

**Figure 2:**
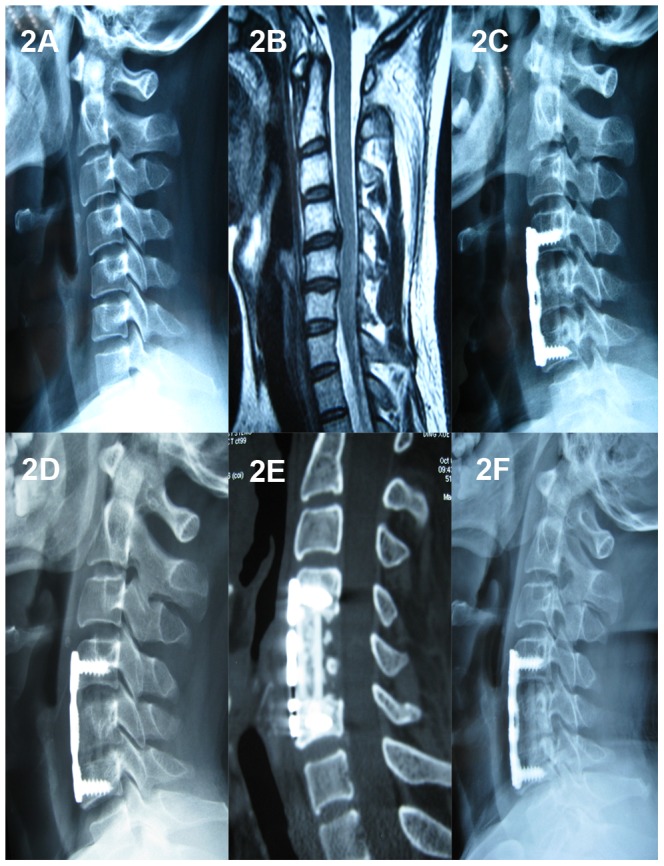
A 36-year-old male who underwent 1-level corpectomy with a nano-hydroxyapatite/polyamide66 cage used for cervical reconstruction. The preoperative cervical X-ray film (2A) and MRI scan (2B) show the spinal cord compression resulting from C4/5 and C5/6 disc herniations. The immediately postoperative lateral X-ray (2C) shows C5 corpectomy and the n-HA/PA66 cage used for reconstruction, and an obvious radiolucent gap can be observed between the cage and the endplates. The lateral X-ray film (2D) shows no obvious radiolucent gap, and the 3D-CT (2E) scan shows the autogenous bone granules filling the cage and achieving bony fusion with adjacent endplates at the 1-year follow-up. A lateral X-ray film (2F) at the final follow-up (four years and eight months) shows satisfying bony fusion and no obvious migration or subsidence.

**Figure 3:**
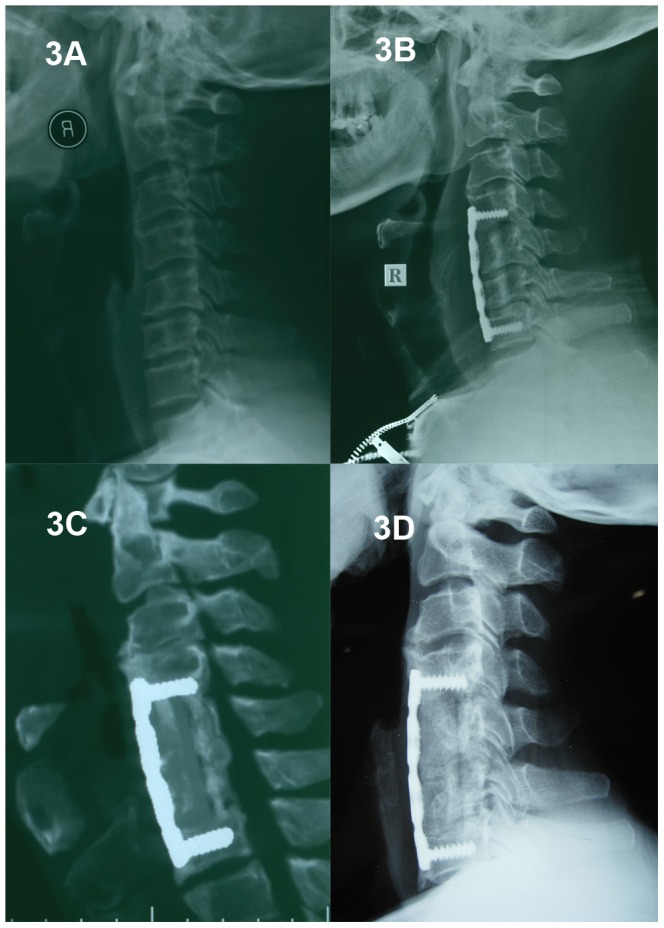
A 61-year-old male who underwent 2-level corpectomy with a nano-hydroxyapatite/polyamide66 cage used for cervical reconstruction. A preoperative cervical X-ray film (3A) shows a loss of cervical lordosis. The immediately postoperative lateral X-ray (3B) shows C5 and C6 corpectomy and the n-HA/PA66 cage used for reconstruction. The 3D-CT (3C) and lateral X-ray (3D) show obvious bony fusion and restoration of cervical alignment at the final follow-up (four years and four months).

**Figure 4:**
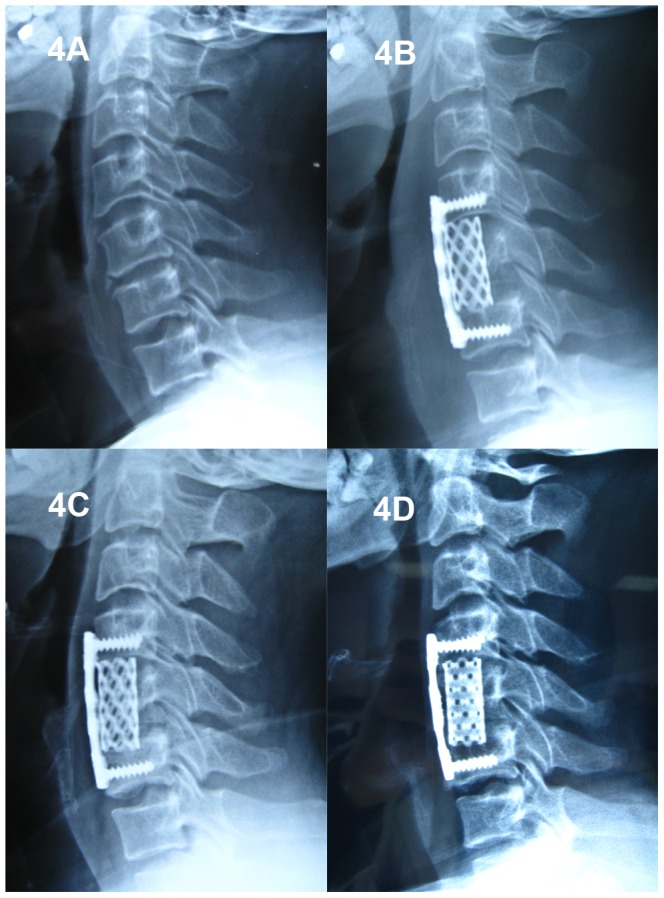
A 53-year-old male who underwent 1-level corpectomy with a titanium mesh cage used for cervical reconstruction. The preoperative cervical X-ray film (4A) and immediately postoperative lateral X-ray (4B) show C5 corpectomy and the titanium mesh cage used for reconstruction. The lateral X-ray one year postoperative (4C) and at the final follow-up (two years and six months; 4D) shows bony fusion between the graft and the adjacent endplates; nevertheless, marked cage subsidence was observed.

**Figure 5:**
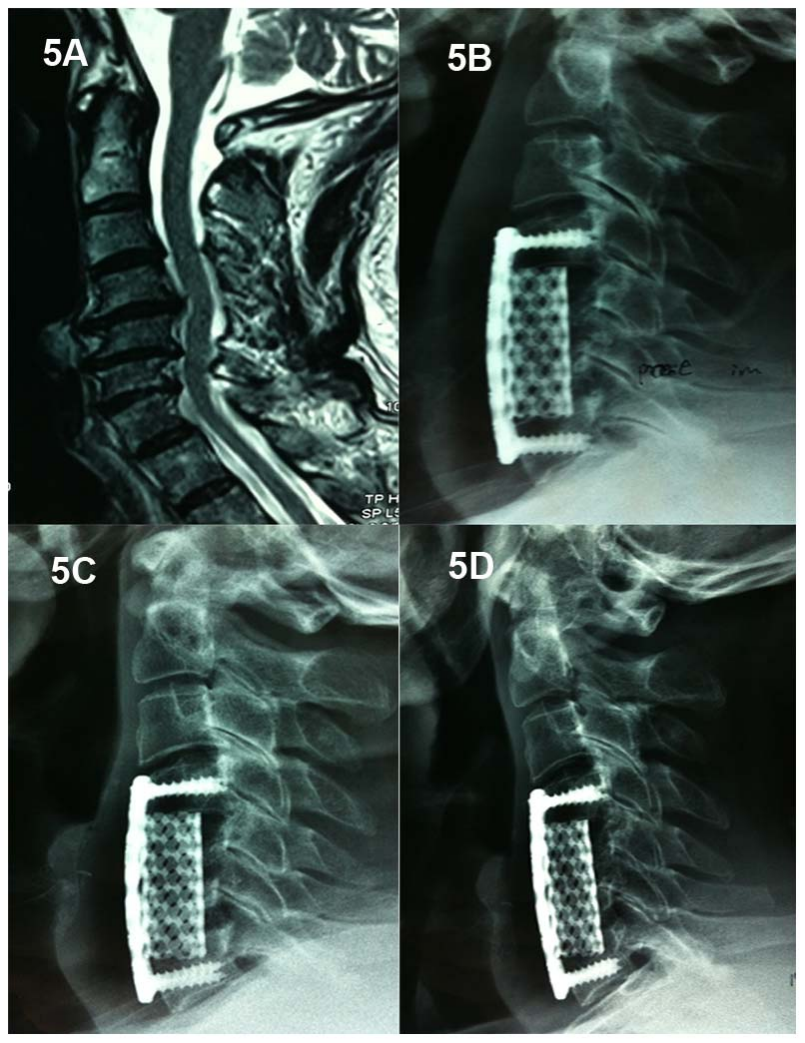
A 46-year-old male who underwent 2-level corpectomy with a titanium mesh cage used for cervical reconstruction. A cervical MRI scan (5A) shows multi-level disc herniations (C4/5, C5/6, C6/7) and oppression of the spinal cord. The immediately postoperative lateral X-ray (5B) shows C5 and C6 corpectomy and the titanium mesh cage used for reconstruction in which the cervical alignment was marginally restored. A lateral X-ray at the one-year follow-up (5C) shows subsidence. A lateral X-ray at the final follow-up (5D) shows increased subsidence and a loss of cervical alignment.

A total of 117 patients (65 males and 52 females) were included in this study, with a mean follow-up of 45.28±12.83 months (range: 25 to 70 months). Based on the number of corpectomies (1- or 2-level) and cage selection (TMC or n-HA/PA66 cage), the patients were divided into four groups. 52 patients underwent 1-level ACCF (Figure 2) and 19 patients underwent 2-level ACCF (Figure 3) with n-HA/PA66 cages. 25 patients underwent 1-level ACCF (Figure 4) and 21 patients underwent 2-level ACCF (Figure 5) with TMCs. The demographics of the patients are shown in Table 1. No significant differences were detected in gender, age, hospital stay, surgery time, blood loss or follow-up (months) between the TMC group and the n-HA/PA66 group for either 1- or 2-level ACCF.

**Table 1:** Demographic and clinical data of patients.

Parameters	1-level ACCF		2-level ACCF	
	TMC	n-HA/PA66 cage	TMC	n-HA/PA66 cage
No.of patients (n)	25	52	21	19
Male/female (n)	11/14	29/23	13/8	12/7
Mean age (years)	55.04±11.09	56.56±12.13	57.81±11.50	57.00±10.95
Hospital stay (days)	16.04±3.67	14.90±3.73	16.76±4.04	15.42±2.39
Surgery time (minutes)	148.40±24.82	143.65±30.50	186.19±28.54	184.74±26.32
Blood loss (ml)	145.20±61.85	133.46±68.57	189.52±90.30	173.68±58.61
Follow-up (months)	49.80±13.06	44.06±13.60	43.42±12.18	44.74±10.33
Involved segments				
1-level corpectomy C4/C5/C6	6/16/3	7/36/9		
2-level corpectomy C4-C5/C5-C6			10/11	11/8

Radiological and clinical parameters are shown in Table 2. For 1-level ACCF, the mean height of the fused segment improved in the TMC group from 52.03±4.35 mm preoperatively to 59.52±4.36 mm immediately postoperative, and it improved from 53.55±6.20 mm to 61.84±6.86 mm in the n-HA/PA66 cage group. Similar results were observed for 2-level ACCF, with heights of 71.71±6.16 mm preoperatively improving to 80.04±6.00 mm immediately post-operative in the TMC group, whereas the heights were 70.30±8.08 mm in the n-HA/PA66 group preoperatively and improved to 77.28±7.56 mm immediately postoperative. Preoperative or immediately postoperative fused segment heights did not differ significantly for either 1- or 2-level ACCF between the TMC and n-HA/PA66 groups.

**Table 2:** Radiographic and clinical outcomes in each group.

Parameter	1-level ACCF			2-level ACCF		
	TMC	n-HA/PA66 cage	P	TMC	n-HA/PA66 cage	P
Height of fused segments (mm)						
Pre-operation	52.03±4.35	53.55±6.20	0.29	71.71±6.16	70.30±8.08	0.52
Immediately post-op	59.52±4.36	61.84±6.86	0.11	80.04±6.00	77.28±7.56	0.21
Loss of height of fused segments (mm)						
One year follow-up	2.13±0.68	1.18±0.58	<0.01	2.63±0.61	1.57±0.58	<0.01
Last follow-up	2.62±0.82	1.56±0.61	<0.01	3.05±0.59	1.88±0.57	<0.01
Fusion rate						
One year follow-up	(22/25) 88%	(49/52) 94%	0.34	(16/21) 76%	(17/19) 90%	0.27
Last follow-up	(24/25) 96%	(51/52) 98%	0.59	(20/21) 95%	(18/19) 95%	0.44
Subsidence rate						
One year follow-up	(4/25)16%	(1/52) 2%	0.02	(7/21) 33%	(1/19) 5%	0.03
Last follow-up	(6/25) 24%	(2/52) 4%	0.01	(8/21) 38%	(1/19) 5%	0.01
C2-C7 Cobb angle (°)						
Pre-op	8.60±5.77	9.69±6.14	0.41	9.33±6.34	9.16±6.81	0.93
Immediately post-op	12.76±5.10	13.15±5.13	0.77	13.10±5.02	13.89±6.39	0.68
Last follow-up	9.96±5.29	10.98±5.20	0.44	9.81±5.81	12.16±6.18	0.23
JOA score (points)						
Pre-operation	12.24±2.18	12.17±2.26	0.87	11.10±2.53	11.63±1.86	0.42
Immediately post-op	14.40±1.63	14.75±1.37	0.41	13.48±2.23	14.26±1.33	0.23
Last follow-up	14.88±1.59	15.37±1.24	0.25	13.76±2.34	14.84±1.83	0.1
VAS of neck pain (points)						
Pre-operation	4.76±1.85	4.37±1.66	0.23	5.14±1.24	5.26±1.59	0.76
Immediately post-op	2.04±1.02	1.85±1.07	0.56	3.00±1.22	2.63±1.16	0.35
Last follow-up	1.44±1.08	1.17±1.25	0.42	2.33±1.06	1.58±1.12	0.06

However, the loss in height of the fused segment in the TMC group was greater than that in the n-HA/PA66 group at the one-year follow-up (2.13±0.68 mm vs. 1.18±0.58 mm, p<0.01) and at the final follow-up (2.62±0.82 mm vs. 1.56±0.61 mm, p<0.01) for the 1-level ACCF, with similar results observed for 2-level ACCF (2.63±0.61 mm vs. 1.57±0.58 mm, p<0.01) at the one-year follow-up and (3.05±0.59 mm vs. 1.88±0.57 mm, p<0.01) at the final follow-up. The TMC group also showed a significantly greater rate of subsidence for 1-level ACCF than the n-HA/PA66 group at one year (16% vs. 2%; p = 0.02) and at the final follow-up (24% vs. 4%; p = 0.01). Furthermore, for 2-level ACCF, the TMC group suffered a more marked incidence of subsidence than the n-HA/PA66 group at one year (33% vs. 5%; p = 0.03) and at the final follow-up (38% vs. 5%; p = 0.01). The majority of cases with cage subsidence showed bony fusion at the final follow-up; moreover, no progression to neurological manifestations arose. No case with subsidence received revisional surgery. Bony fusions of the grafts were similar at the final follow-up, with 96% (24/25) of patients in the TMC group exhibiting them vs. 98% (51/52) in the n-HA/PA66 group for 1-level ACCF, whereas for 2-level ACCF, 95% (20/21) of patients developed bony fusions in the TMC group vs. 95% (18/19) in the n-HA/PA66 group. However, at the one-year follow-up, the TMC group exhibited a lower rate of bony fusion, although not statistically significant, than the n-HA/PA66 group (22/25 (88%) vs. 49/52 (94%) for 1-level ACCF; 16/21 (76%) vs. 17/19 (90%) for 2-level ACCF). No revisional surgery was required for patients who did not exhibit bony fusion at the final follow-up because the anterior cervical plate and screws remained in position and the patients did not complain of discomfort. For all four groups, there was a slight improvement in the mean C2–C7 Cobb angle when preoperative values were compared with the final follow-up measurements. When preoperative or final follow-up postoperative measurements were compared, no significant differences were detected in Cobb angle between the TMC and n-HA/PA66 groups either for 1- or 2- level ACCF.

The preoperative JOA scores did not differ between the TMC and n-HA/PA66 groups regardless of the number of levels fused. At the final follow-up, JOA scores had improved significantly in all four groups. No significant differences were detected between the TMC and n-HA/PA66 groups for either 1-or 2-level ACCF at the final follow-up. The mean preoperative VAS score was similar between the two kinds of cage groups for both 1- and 2-level ACCF. However, at the last follow-up, the mean VAS score in the TMC group was higher, albeit insignificantly, than that of the n-HA/PA66 group (1.44±1.08 vs. 1.17±1.25 (p = 0.42) for 1-level ACCF; 2.33±1.06 vs. 1.58±1.12 (p = 0.06) for 2-level ACCF).

## Discussion

In recent years, ACCF has been recognized as a reliable and effective procedure for the treatment of MCSM. The advantage of the direct decompression resulting from the resection of the object causing oppression of the spinal cord from the anterior column is supported by the many reports of successful clinical outcomes in treating MCSM [26], [27]. In addition to decompression, reconstruction of the cervical spine is a critical procedure. TMC has been used widely for years. Packed with autogenous graft from the removed vertebra, this apparatus can provide early biomechanical stabilization of the anterior column, restoration and maintenance of the intervertebral height and cervical alignment and enlargement of the stenotic neural foramen and can avoid the potential complications caused by autogenous graft collection [28]. However, implant-related complications cannot be ignored. TMC subsidence, the typical hardware-related complication, has been reported to range from 0% to 30% [29]. Although the relevance of TMC subsidence remains controversial, the subsidence can have serious consequences, such as the buckling of the cervical ligamentum flavum, foraminal stenosis and re-compression of the cervical spinal cord and nerve roots [16], [17], [28], [29].

The n-HA/PA66 is a composite made by infiltrating nano-HA into PA66; it mimics natural bone in that apatite is distributed within a collagen matrix. Thus, the composite possesses both the mechanical strength of HA and the elastic properties of PA66. A previous study documented that the n-HA/PA66 composite was safe and that its mechanical properties complement natural bone well [22]. The n-HA/PA66 cage is a biomimetic implant fabricated from n-HA/PA66 composite. This device has been used for spinal reconstruction for several years, and considerable clinical results have been reported [20], [21], [30]. Our previous research showed a 94.3% bony fusion rate in 35 patients and a 2.9% subsidence rate with n-HA/PA66 cages in reconstructions following cervical corpectomy [20]. Yang et al. [30] reported using n-HA/PA66 cages for anterior reconstruction after thoracolumbar corpectomy in 51 patients and achieved a 90.2% bony fusion rate with a low incidence of cage subsidence. Yang et al. [21] achieved a 97% fusion rate and a 6% subsidence rate in 35 patients with n-HA/PA66 cages for single-level cervical corpectomy and fusion.

Subsidence was typically observed when TMC was used for cervical reconstruction after corpectomy. Daubs et al. [31] described an early subsidence in 30% (7 of 27) of cases with ACCF. In our series, by the final follow-up, for 1-level ACCF, we observed that the n-HA/PA66 group exhibited a significantly lower rate of subsidence than the TMC group (4% vs. 24%; p = 0.01). Moreover, the difference in subsidence rates was much greater when compared for 2-level ACCF, we observed subsidence in 8 of 21 cases (38%) in the TMC group vs. 1 of 19 cases (5%) in the n-HA/PA66 group (p = 0.01). Increased patient age, severe osteoporosis, excessive endplate removal and intra-operative over-distraction have been demonstrated to be risk factors of TMC subsidence [17]. However, the metal attributes and the shape of the TMC are likely the most important factors. The sharp teeth at both ends of the TMC are beneficial to early stabilization by embedding the cage into adjacent endplates. Unfortunately, the contact interface between the TMC and endplates is small (we describe this pattern as a “point-to-surface contact”), and the intensity of pressure at the contact surface is so great that it may result in excessive insertion of the cage into the vertebra. In contrast, the n-HA/PA66 cage has a broader surface at both ends with which to contact the endplates. We describe this pattern as “surface-to-surface contact,” and it distributes the loads on the interface and decreases incidences of cage subsidence. Unlike the sharp teeth that the TMC possesses, the n-HA/PA66 cage has grooves at the terminal faces that can increase the friction between the cage and endplates and that is sufficient to prevent cage migration. In our study, even in the case of 2-level ACCF, we did not observe n-HA/PA66 cage migration or extrusion.

Majd ME et al. [32] reported a fusion rate of 97% in cervical reconstructions with TMC, and a fusion rate of 100% was observed by Rieger et al. [33] using the same technique. In our study, for both 1- and 2-level ACCF, the fusion rates in the TMC group and the n-HA/PA66 group were almost equivalent at the final follow-up. However, the n-HA/PA66 group showed higher fusion rates, albeit insignificantly, than the TMC group at the 1-year follow-up (94% vs. 88% (p = 0.34) for 1-level ACCF; 90% vs. 76% (p = 0.27) for 2-level ACCF). Previous reports have demonstrated that the difference in fusion conditions was primarily due to the different elastic moduli between the two struts [20], [22]. Compared with TMC, the n-HA/PA66 cage possesses an elastic modulus similar to that of the autogenous graft inside the cage [22], [34]. As described by Wolff's law, bone grows in response to applied stress and is resorbed when mechanical stimulus is absent. Due to the appropriate elastic modulus, n-HA/PA66 cages may reduce stress shielding and promote fusion effectively. In addition, the n-HA/PA66 cage exhibits excellent biocompatibility and osteoconductive ability in vivo. Animal experiments demonstrated that when implanted, the cage can release Ca^2+^ and PO_4_^3−^ from its surface, which gradually forms a crystal layer on the cage surface that bridges the graft and implant bed to provide a trestle for osteogenesis [35]. Moreover, the holes in the n-HA/PA66 cage wall may enable blood circulation between the implant bed and the autograft within the cage, which would aid in the growth of the bone graft. Previous studies have reported that subsidence occurs in up to 80% of patients during the early postoperative period prior to bony fusion [36]. In the present study, similar results were observed in all four groups, with the majority of height loss in the fused segment occurring during the 1^st^ postoperative year and heights remaining almost identical to these levels at the final follow-up. Considering the reduced loss of fused segmental height and earlier bony fusion in the n-HA/PA66 group compared with the TMC group, the data indicate that the earlier bony fusion in the n-HA/PA66 group may contribute to the decrease in the loss of fused segmental height. However, the differences in present study are still insignificantly. In the future, larger samples and longer follow-ups should be required to demonstrate this conclusion.

Some studies have demonstrated that the number of corpectomy levels is a risk factor for TMC subsidence. The increase in ACCF levels may eventually cause not only a higher incidence of subsidence but also more severe subsidence when TMC cages are used [31], [37]. We observed similar outcomes in our TMC groups. As a result of these published data, previous reports have suggested that 2-level ACCF using TMC cages may not be appropriate therapeutic options for treating MCSM. However, in this study, even for 2-level corpectomy, we observed that the amount of subsidence was much lower in the n-HA/PA66 group compared with the TMC group. Furthermore, we did not observe a high incidence of subsidence. These observations suggest that the n-HA/PA66 cage may provide greater stability than the TMC and may more effectively maintain fused segmental height. The data also indicate that the n-HA/PA66 cage may be a better therapeutic choice for 2-level ACCF.

With the utilization of TMC in cervical reconstruction after corpectomy, restoration of cervical alignment is possible [24]. In the present study, we employed the C2–C7 Cobb angle to evaluate cervical sagittal alignment, the preoperative Cobb angle were similar in four groups, and we observed an improvement in the Cobb angle at the final follow-up compared with preoperative values in all four groups, respectively. There were no significant differences in the Cobb angle at the final follow-up between the TMC and n-HA/PA66 groups in both the 1- and 2-level ACCF; however, we noted that the n-HA/PA66 groups showed little better improvements of C2–C7 Cobb angles. These differences may be due to the greater loss in segmental height when using TMC for cervical reconstructions.

The JOA and VAS scales were used to assess clinical efficacy in our study. We noted improvements in these two parameters in all four groups. At the final follow-up, the mean JOA score was similar between the TMC and n-HA/PA66 groups for both the 1- and 2-level ACCF. However, we observed that the patients in the TMC group presented with higher, albeit non-significant, VAS scores than those in the n-HA/PA66 group, particularly in the 2-level ACCF group. Previous studies [9], [31], [36], [37] showed that subsidence was strongly correlated with neck pain. Although no significant difference was detected in this outcome in our study, these results nevertheless indicate that patients treated with n-HA/PA66 cage for ACCF may suffer less axial neck pain than patients treated with TMC. In our view, the increased loss in segmental height in the TMC group may underlie these differences, and we will pay close attention to this issue in future follow-ups.

## Conclusion

This retrospective study demonstrated that both the TMC and n-HA/PA66 cage resulted in effective clinical and radiographic outcomes when used to treat MCSM with cervical reconstruction after corpectomy. However, with its optimized biomechanical characteristics and unique shape, the n-HA/PA66 cage achieves similar bony fusion rates but lower rates of subsidence. Furthermore, even in the case of 2-level ACCF, the n-HA/PA66 cage can maintain fused segment height better and lower incidences of subsidence compared with TMC. The n-HA/PA66 cage may be a better alternative for cervical reconstruction than TMC after corpectomy, particularly for 2-level ACCF.
